# Adoption of Digital Technologies in Public Health Crises: An International Large‐Scale Survey

**DOI:** 10.1111/phn.70073

**Published:** 2026-01-30

**Authors:** Laura‐Maria Peltonen, Zerina Lokmic‐Tomkins, Hwayoung Cho, Emma Collins, Hanna von Gerich, Christoph Golz, Michelle Honey, Tamara G. R. Macieira, Maxim Topaz, Dawn Dowding

**Affiliations:** ^1^ Department of Health and Social Management University of Eastern Finland Kuopio Finland; ^2^ Turku University Hospital Turku Finland; ^3^ Kuopio University Hospital Kuopio Finland; ^4^ School of Nursing and Midwifery and Climate and Health Initiative Monash University Melbourne Australia; ^5^ Department of Family Community and Health System Science College of Nursing University of Florida Gainesville Florida USA; ^6^ Department of Nursing University of Otago Dunedin New Zealand; ^7^ Department of Nursing Science University of Turku Turku Finland; ^8^ School of Health Professions Bern University of Applied Sciences Bern Switzerland; ^9^ School of Nursing The University of Auckland Auckland New Zealand; ^10^ School of Nursing and Data Science Institute Columbia University New York USA; ^11^ Division of Nursing Midwifery and Social Work School of Health Sciences University of Manchester Manchester UK

## Abstract

**Objectives:**

To examine nurses’ adoption and use of digital technologies across six countries during the COVID‐19 pandemic and identify lessons to strengthen preparedness for future public health crises.

**Methods:**

Nurses in Australia, Finland, New Zealand, Switzerland, the United Kingdom and the United States completed an international cross‐sectional survey in 2022–2023. Recruitment used snowball sampling via professional networks, associations and social media. The 41‐item questionnaire captured information on technologies adopted during the pandemic, their perceived usability and contextual factors influencing their implementation.

**Results:**

In total, 1,423 nurses reported on 1,128 technologies. Usability varied across countries and technology categories, with average System Usability Scale (SUS) scores at the benchmark for average usability. Some countries reported higher usability than others, suggesting differences in digital infrastructure maturity and workflow integration. Across settings, respondents described challenges related to digital literacy and skills, technical barriers and connectivity, organizational readiness, training, usability and accessibility, as well as dependency on technology. These influenced adoption and effective use during the pandemic.

**Conclusions:**

Nurses’ experiences revealed variations in usability and implementation challenges, demonstrating that nurses were underprepared for rapid digital transformation. Strengthening digital literacy, technical infrastructure and organizational readiness supports safe and effective technology integration in future public health crises.

## 1 Introduction

The COVID‐19 pandemic disrupted healthcare systems worldwide, necessitating the adoption of innovative approaches to provide safe and effective patient care. Nurses were obligated to rapidly adopt many new technologies to provide care (Dykes and Chu 2021). During the early stages of the pandemic, technology shaped care through (1) interdisciplinary networks for robust platforms for disease monitoring, (2) collaboration between professionals, patients, families and industry and (3) exploitation of social media and health applications to reach different stakeholders (Atique et al. 2020). Later, telehealth and eHealth solutions became considered convenient, safe, scalable, sustainable and effective methods of providing clinical care during this public health crisis (Bokolo 2021). The nurses’ work quickly transformed to embed virtual consultations over the telephone, smart tablet or video conferencing (Meehan and Honey 2020), ultimately transforming the interaction between nurses and patients and families (Cadel et al. 2021).

The pandemic transformed care delivery through technology adoption, such as increasing remote patient monitoring that allows continuous patient monitoring while reducing the exposure risk for nurses (Santos et al. 2021). These technologies were adopted throughout the patient care pathway within hospitals (Santos et al. 2021), during the transition of care (Patel et al. 2022) and at home (Blöndal et al. 2022). The pandemic also drove a rapid adoption of virtual learning and training solutions in nursing education and continuous learning (Hargreaves et al. 2021, Jeon et al. 2024). Online nursing education ensured the continuity of education during social distancing and facilitated knowledge sharing and collaboration among healthcare professionals (Pozzi et al. 2023). Adopting different digital technologies to support learning also allowed for more accessible, flexible and personalized learning opportunities in the long‐term (Pang et al. 2023).

Understanding why some technologies were successfully adopted while others were not requires a structured theoretical perspective. The Non‐Adoption, Abandonment, Scale‐up, Spread and Sustainability (NASSS) ‐framework provides a useful theoretical lens for better understanding the complexities underlying such rapid technology adoption. This framework helps examine the complexities influencing technology adoption across seven domains over time, including the treated condition, the adopted technology and its proposed value, the technology users and their organization, and the wider political, regulatory, professional and sociocultural system (Greenhalgh et al. 2017). Instead of overlooking or oversimplifying the complexities affecting technology adoption, the NASSS‐framework encourages acknowledging, addressing and mitigating them (Greenhalgh and Abimbola 2019). It has been successfully used to guide the implementation of a variety of digital technologies, including telemedicine, consumer health technologies and decision support systems (Shin et al. 2025). Examples highlighting the value of using the NASSS‐framework to examine the factors affecting technology adoption include literature reviews on computerized clinical decision support systems (Abell et al. 2023), video consulting systems adopted during the COVID‐19 pandemic (James et al. 2021) and eHealth solutions for people with intellectual disabilities (van Calis et al. 2023).

The NASSS‐framework also helps explore organizational resilience and response in critical or unexpected circumstances (Greenhalgh et al. 2017), highlighting its applicability in studying technology adoption during healthcare crises like the COVID‐19 pandemic. Previous studies indicate that while the rapid global transformation and adoption of technologies during the pandemic presented many opportunities, it concurrently created risks for inappropriate technology adoption (Meinert et al. 2018). Specific issues related, for example, to technology access, workload, hybrid working, disruption to therapeutic relationships, safety risks and lack of involvement in decision‐making were identified (Anderson et al. 2024). The complex contexts and mechanisms related to implementing new technologies in healthcare are associated with nurses' work motivation, engagement, satisfaction and well‐being (Jedwab et al. 2023). The pandemic underlined the importance of developing approaches and guidelines that support safe, rapid technology adoption, emphasizing the need to include nursing perspectives and input in the process (Vasilica et al. 2023). It also prompted health policymakers to reassess national digital health strategies, including improving perspectives on usability, interoperability, data management, privacy, security, and digital inclusivity (Sheikh et al. 2021).

Broader global trends further reinforce the urgency of strengthening system preparedness. Rising global temperatures increase the likelihood of climate‐related disasters and future pandemics, highlighting the need for resilient health systems capable of adapting quickly. The World Health Organization (WHO) has published a framework to guide healthcare organizations in preparing for future disasters (World Health Organization 2015). This framework includes ten key components for health organizations, authorities and institutions to mitigate, adapt and increase resilience to climate‐related health risks. These components include strengthening the necessary infrastructure and technologies to improve healthcare performance, applying leadership and governance to prioritize and protect integral policies and strategies, and building evidence to support decision‐making through health and climate research. Part of this preparedness is the ability to adapt and transform services rapidly and learn from experience to improve system capacity in the future. Although there is research on adopting technologies in particular countries during the pandemic (Bokolo 2021, Cadel et al. 2021, Santos et al. 2021, Patel et al. 2022, Blöndal et al. 2022, Hargreaves et al. 2021, Anderson et al. 2024, Vasilica et al. 2023), cross‐national research is needed to understand the similarities, differences and best practices in adopting digital technologies in nursing during public healthcare crises. Additionally, the existing evidence often focuses more on implementing and using specific technologies rather than evaluating their broader impact on patient outcomes or nursing practice (Wieben et al. 2023).

This study is part of an international collaboration that provides an opportunity to follow up and extend earlier work by nurse informaticians, who have explored nurses’ use of technology during the early phases of the pandemic (Collins and Honey 2021, Grindle 2021, Logsdon 2022, 2022). A cross‐national comparative approach was adopted to move beyond country‐specific assumptions and settings, generating insights transferable across diverse healthcare systems to inform better‐informed healthcare practice, research and policy (Heath et al. 2024). Guided by the NASSS framework, this study aimed to examine the digital technologies adopted by nurses during the COVID‐19 pandemic across six countries, focusing on their usability and adoption challenges, and to identify lessons learned to support future public health crisis preparedness and response.

## 2 Methods

### 2.1 Design

This descriptive cross‐sectional and cross‐national survey study was undertaken in 2022–2023 to address the following research questions:

1. Which technologies have been adopted in nursing to support health care globally during the pandemic?
2. What experiences do nurses have regarding the usability of these technologies?
3. What factors are associated with technology adoption and use?
4. What cross‐national patterns emerge from the results, and how might these shared trends guide policy‐level decision‐making for future public health crises?

### 2.2 Setting and Population

This international project targeted registered nurses working during the COVID‐19 pandemic across six countries. Participant recruitment was carried out separately in each participating country using a snowball sampling technique. The survey was conducted without limiting the number of participants. Invitations to participate anonymously in the self‐administered online survey were distributed via the professional networks of the researchers and through national professional associations (e.g. nurses’ associations) and social media (LinkedIn, Facebook and X). Specifically in the USA, to recruit a broad sample of registered nurses, the study team distributed the recruitment email and flyer using the State Board of Nursing registry lists. This broad, national distribution resulted in a higher number of registered nurses reached relative to other participating countries. The participants were not reimbursed for their involvement in the study.

### 2.3 Questionnaire Development

The questionnaire was initially designed by the UK members of this international collaboration (Dowding et al. 2023) as an online questionnaire for nurses. It sought feedback on technologies implemented in healthcare services during the pandemic. Developed by nurses, academics and two patient and public representatives, the questionnaire was guided by the NASSS‐framework (Greenhalgh et al. 2017), incorporating key details related to the different domains affecting technology adoption. After iterative feedback and pilot testing with 10 UK nurses in digital and informatics roles, revisions were made to the final version.

The final 41‐item survey included questions on demographics, adopted digital technologies and an optional section on nurses’ attitudes toward health technology. Respondents could report on up to three technologies. Following the NASSS‐framework, the questions explored the different domains of technology adoption, including system integration, maturity, target users, technology features, technology training and support, perceived usability and possible concerns expressed by the patient. The survey included multiple‐choice, net promoter rating, and free‐text questions. The survey also included the SUS (https://www.usability.gov/how‐to‐and‐tools/methods/system‐usability‐scale.html), a 10‐item measure of technology usability. The original English version was used in English‐speaking countries, while researchers with nursing informatics expertise translated Finnish and German adaptations. Local clinicians and researchers assessed face validity.

For the purpose of this international study, selected items of the survey were chosen for comparison. These questions included:

- *What does the technology do? For example, Monitor patient's heart rate, blood pressure, etc*. [free‐text]
- *Please rate this technology using the SUS below* [multiple choice]
- *Has the pandemic highlighted problems or glitches with frequently used technology? Please describe any factors you think are relevant* [free‐text]

Other items, including items assessing nurses’ attitudes toward digital technologies, were not included in this cross‐national analysis, as they will be reported separately in national‐level publications and fell outside the predefined scope of this study.

### 2.4 Data Analysis

Following the inductive content analysis methodology (Elo and Kyngäs 2008), the free‐text responses to open‐ended questions were coded and organized into thematic categories. The main categories were then quantified and analyzed using descriptive statistics (*n*, %) to allow cross‐national comparison. Similarly, responses to closed questions were analyzed using descriptive statistics (*n*, %).

The overall SUS scores were calculated for each main technology category. Initially, the overall SUS scores were computed following Brooke's (1996) method (Brooke 1996). The scale was converted into numbers (from 1 = strongly disagree to 5 = strongly agree), X = sum of scores for all odd‐numbered questions ‐5; Y = 25‐sum of scores for all even‐numbered questions. The overall SUS Score = (X + Y) × 2.5, resulting in a scale from 0 to 100. Average SUS scores were then calculated for each technology category to identify those with higher usability. The literature suggests that an overall SUS score of 68 is a benchmark, with half of the usability assessments falling below and above it, indicating average usability. In contrast, scores above 80.8 are above average and scores below 50 indicate poor usability (Sauro and Lewis 2016, Lewis and Sauro 2018). Technology counts and percentage representation within each category were also computed for distribution analysis. We explored differences in the overall SUS scores between countries and main technology categories with the Kruskal–Wallis Test. Dunn's post‐hoc test was conducted to identify statistically significant pairwise differences in overall SUS scores across countries, following the Kruskal–Wallis test. Statistical computations and data management were done using Python (version 3.11.4; Python Software Foundation).

### 2.5 Ethical Review

Each country was responsible for ethical and administrative approvals before survey distribution. Ethical review was handled in each country separately, including Australia (Monash University, HREC 35511), Finland (University of Turku Ethics Committee for Human Sciences, 39/2022), New Zealand (University of Otago Ref: 22/070; Ngāi Tahu Research Consultation Committee Ref: 6043_23423), and the USA (University of Florida, IRB202202164). The UK study was exempt from ethics, and the Swiss Human Research Act did not apply to the study according to the Swiss Federal Act on Research Involving Human Beings (see 810.30 HRA Art. 2) since data were collected anonymously and no health‐related data were included.

## 3 Results

### 3.1 Characteristics

The respondents (*n* = 1423 nurses, see Table 1 for characteristics) described and rated the usability of 1128 technologies, including those from Australia *n* = 92, Finland *n* = 84, New Zealand = 149, Switzerland = 146, the United Kingdom = 81, and the USA = 576. The technology descriptions were grouped by the main technology functionality, resulting in 12 main technology categories. The most reported technology categories across countries were related to (1) care and patient information (*n* = 258), (2) monitoring (*n* = 240), (3) telehealth (*n* = 155), (4) professional knowledge exchange (*n* = 137) and communication (*n* = 111). The categorization of technologies with frequencies is presented in Table 2.

**TABLE 1:** Characteristics of respondents.

	N	%
Country		
Australia	106	7.45
Finland	55	3.87
New Zealand	203	14.27
Switzerland	171	12.02
United Kingdom	136	9.56
United States of America	752	52.85
Total	1423	100
Type of organization		
Primary care/community	376	26.42
Secondary/tertiary care	763	53.62
Other	150	10.54
Missing	134	9.42
Total	1423	100
Role of respondent		
Staff nurse, midwife, public health nurse	670	47.08
Advanced nursing role	184	12.93
Academic, educator	44	3.09
Informatics (incl. leadership, e.g., CNIO)	31	2.18
Leadership	239	16.80
Other	34	2.39
Missing	221	15.53
Total	1423	100
Respondent in a management role		
Yes	372	26.14
No	844	59.31
Missing	207	14.55
Total	1423	100
The organization has a dedicated team responsible for the implementation of digital solutions in nursing		
Yes	656	46.10
No	304	21.36
Do not know	330	23.19
Missing	71	4.99
Total	1423	100

**TABLE 2:** Categorization of technologies grouped by main function.

Category	Definition	Examples	Count	%
1. Care and patient information systems	Digital systems used to document, retrieve and manage patient information essential for clinical care and medication processes.	Electronic health records (EHRs), lab label printer, care information system, e‐prescription	258	22.87
2. Monitoring equipment	Devices that continuously or intermittently track patients’ physiological parameters to support clinical decision‐making.	Vital‐sign monitors, remote pulse oximeters, cardiac telemetry devices, wearable monitoring sensors	240	21.28
3. Telehealth solutions	Technologies enabling remote patient assessment, consultation, and communication through synchronous or asynchronous platforms.	Video‐consultation platforms, telephone triage systems, digital tools for remote follow‐up	155	13.74
4. Professional knowledge exchange systems	Platforms that support collaboration, communication and knowledge sharing among healthcare professionals.	Online meeting and shared document platforms, digital handover tools	137	12.15
5. Communication systems	Tools used to facilitate communication between staff or between staff and patients, often supporting day‐to‐day coordination.	Smartphones, secure messaging apps, tablets used for patient‐family video connection	111	9.84
6. Data management infrastructure	Back‐end systems enabling access, flow and storage of clinical data across locations, organizations or devices.	Remote access to EHRs, inter‐organizational data‐sharing platforms, secure VPN connections	47	4.17
7. Medical devices	Hardware used in clinical assessment, diagnosis or treatment, often integrating digital functionalities.	Infusion pumps, ventilators with digital interfaces, glucometers, diagnostic scanners.	44	3.90
8. Covid specific systems	Technologies developed or deployed specifically to respond to COVID‐19–related clinical and organizational needs.	COVID‐19 testing devices, symptom‐tracking apps, digital contact‐tracing systems	37	3.28
9. Unclear purpose	Technologies for which respondents provided insufficient detail to assign them reliably to another functional category.	Vague references such as “a tool we used at work” or systems described only by nickname without purpose	32	2.84
10. Knowledge management systems	Platforms supporting education, continued learning and access to evidence or guidelines for clinical practice.	Online learning systems, e‐learning modules, digital training portals	28	2.48
11. Administration, leadership and management systems	Tools supporting operational, managerial and administrative tasks within healthcare organizations.	Workforce allocation tools, shift‐planning systems, workflow management dashboards, office productivity software	22	1.95
12. Digital care pathways and patient portals	Systems that enable patients to access their health information, complete self‐assessments or follow structured care pathways online.	Patient portals linked to EHRs, digital symptom self‐assessment tools, online follow‐up pathways for chronic conditions	17	1.51
Total			1128	100

### 3.2 Usability

The average of the overall SUS scores across countries and technology categories was at the benchmark for average usability, 68.06 (*n* = 1128). We found differences between the overall SUS scores reported in different countries (*H* = 12.30, *p* = 0.031). Dunn's post hoc tests showed that the only difference observed was between Australia and Switzerland (*p* = 0.023). Respondents from Switzerland (71.27) reported the highest scores on average, followed by New Zealand (70.17), the United Kingdom (69.94), Finland (68.39) and the USA (67.20), while Australia (62.99) reported lowest scores overall.

Finland and Switzerland reported the highest overall SUS scores across technology categories. Finland had the highest numbers in knowledge management (95.00) and digital care pathways and patient portals (79.00) while Switzerland had high scores in the ƒcategories on data management infrastructures (92.5) and administration, leadership and management (81.25). In the United Kingdom, the highest score was given to one technology, which belonged to the digital care pathways and patient portals category (SUS 100), and the medical device category followed with an overall SUS score of 91.25. Australia had lower scores than other countries. Australia had lower usability scores than other countries, with the lowest scores in care and patient information systems (55.00) and monitoring (59.09). The average of the overall SUS scores for different technology categories, with the number of reported technologies per each category in brackets, are found by country in Table 3.

**TABLE 3:** The average of the overall system usability scale (SUS) scores for different technology categories, with the number of reported technologies in brackets, as reported by respondents from Australia, Finland, New Zealand, Switzerland, the United Kingdom and the USA.

Main category	Australia SUS (*n*)	Finland SUS (*n*)	New Zealand SUS (*n*)	Switzerland SUS (*n*)	UK SUS (*n*)	USA SUS (*n*)	Total
Administration leadership and management	87.50 (1)	58.75 (2)	73.00 (5)	81.25 (2)	30.00 (2)	67.75 (10)	66.82 (22)
Care and patient information systems	55.00 (15)	71.36 (11)	67.01 (41)	71.13 (84)	75.00 (15)	60.73 (92)	66.07 (258)
Communication	65.56 (9)	72.19 (8)	76.72 (16)	66.35 (13)	68.65 (13)	70.14 (52)	70.25 (111)
COVID19‐specific system	61.25 (2)	65.83 (3)	70.50 (10)	52.50 (2)	71.25 (2)	61.94 (18)	64.53 (37)
Data management infrastructure	61.00 (5)	58.12 (4)	57.29 (12)	92.50 (1)	49.17 (3)	64.55 (22)	61.38 (47)
Digital care pathways and patient portals	68.33 (3)	79.00 (5)	81.25 (2)	0 (0)	100.00 (1)	67.50 (6)	74.56 (17)
Knowledge management	60.77 (13)	95.00 (1)	65.50 (5)	0 (0)	57.50 (1)	68.75 (8)	65.00 (28)
Medical Device	62.50 (2)	0 (0)	77.50 (2)	68.64 (11)	91.25 (2)	64.44 (27)	67.22 (44)
Monitoring	59.09 (11)	76.79 (14)	81.00 (10)	75.00 (3)	75.19 (13)	71.94 (189)	72.23 (240)
Professional knowledge exchange	68.96 (12)	67.22 (18)	67.93 (29)	74.44 (27)	66.67 (6)	69.00 (45)	69.51 (137)
Telehealth	66.25 (18)	67.00 (10)	78.65 (13)	76.25 (2)	68.12 (20)	66.14 (92)	67.65 (155)
Unclear purpose	75.00 (1)	48.75 (8)	68.75 (4)	65.00 (1)	72.50 (3)	51.67 (15)	56.17 (32)
Total	62.99 (92)	68.39 (84)	70.17 (149)	71.27 (146)	69.94 (81)	67.20 (576)	68.06 (1128)

We also found a difference in the overall SUS scores reported for the twelve main categories of implemented technologies (*H* = 41.09, *p* < 0.001). Post hoc tests showed that the most significant differences were observed between the unclear purpose category and monitoring (*p* < 0.001), communication (*p* = 0.004), professional knowledge exchange (*p* = 0.013). Differences were also found between monitoring and data management infrastructure (*p* = 0.011) and care and patient information systems (*p* = 0.035). The highest overall SUS score was seen for the digital care pathways and patient portals category (74.56) while the lowest score was given to the unclear purpose category (56.17).

**FIGURE 1:**
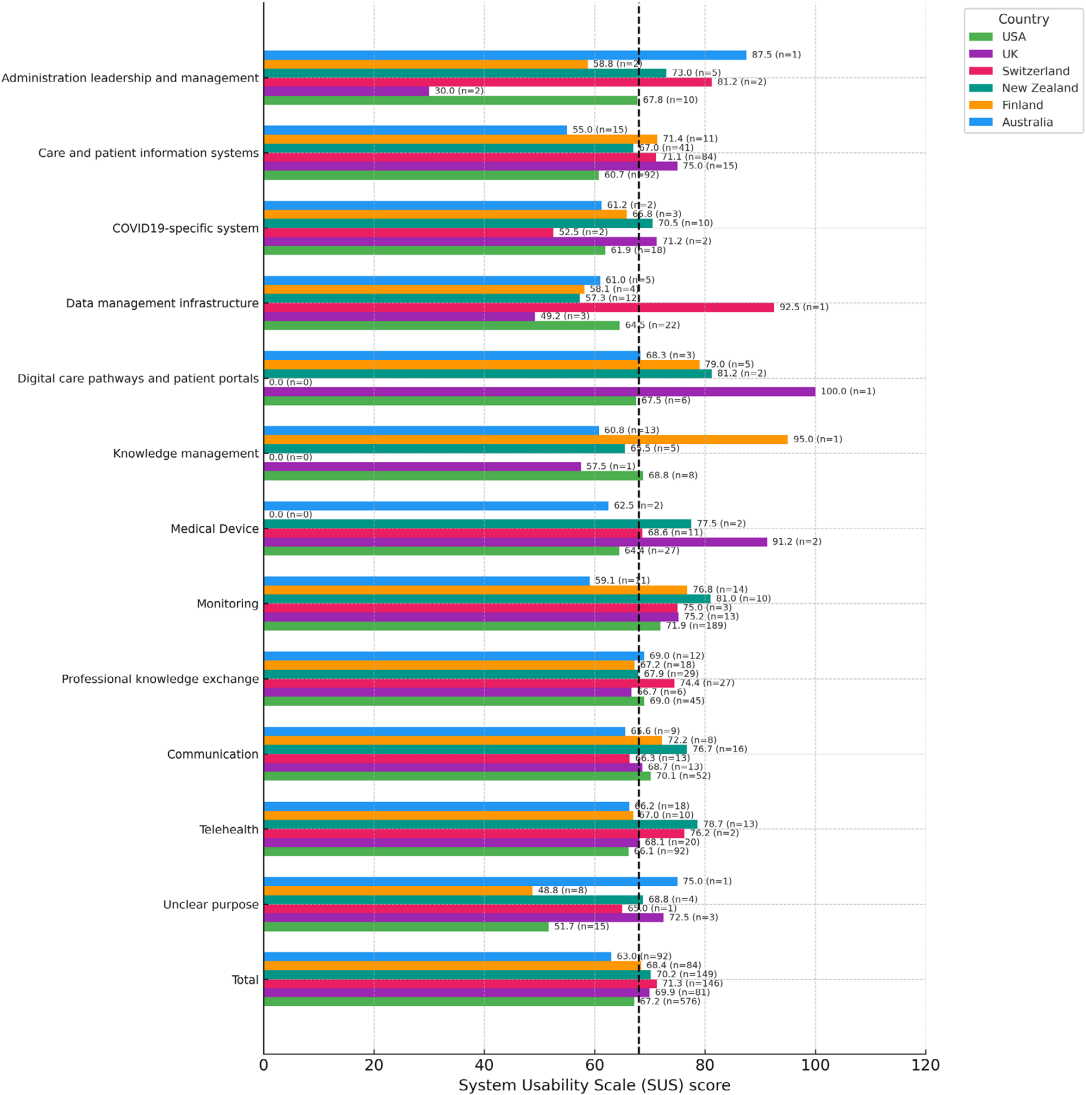
The average of the overall system usability scale (SUS) scores per country and technology category with a line indicating the industry average of 68. [Colour figure can be viewed at wileyonlinelibrary.com]

SUS scores are presented for the identified technology categories, as reported by respondents from six different countries. The scores indicate the perceived usability of these technologies, ranging from 0 to 100, where a higher score reflects better usability. Figure 1 illustrates the variation in SUS scores across technology categories and countries, with labels showing both the mean SUS score and the number of technologies reported (*n*). While most categories cluster around the benchmark for average usability (SUS = 68), notable cross‐national differences are visible, particularly in data management infrastructures, medical devices and digital care pathways and patient portals.

Viewed through a usability lens, these results help explain the range of adoption challenges reported by nurses. As the next section shows, difficulties with skills, workflows, training and system integration often mirrored the usability patterns observed across categories and countries.

### 3.3 Technology Adoption Challenges

**FIGURE 2:**
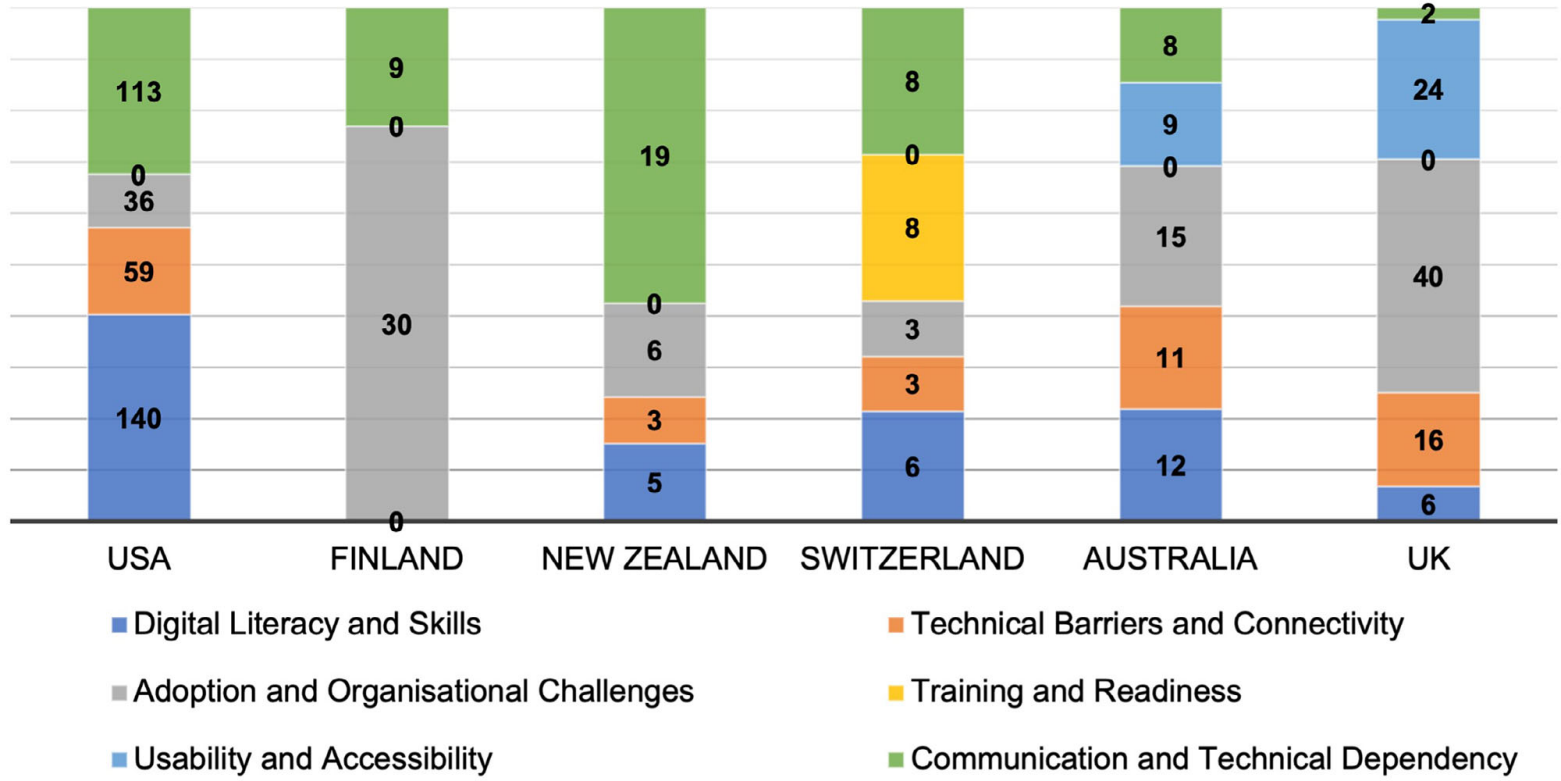
Issues associated with technology adoption are displayed, with counts for each category broken down by country. [Colour figure can be viewed at wileyonlinelibrary.com]

The responses to the question on challenges with implemented technologies yielded 591 unique challenges related to technology adoption across all countries (Australia = 55, Finland = 39, New Zealand = 33, Switzerland = 28, United Kingdom = 88, USA = 348). These challenges were thematically categorized into six main categories: (1) digital literacy and technical skills (e.g. difficulty navigating unfamiliar systems), (2) technical barriers and connectivity (e.g. system slowdowns and unreliable internet connections), (3) adoption and organizational challenges (e.g. technologies were introduced without clear workflows or organizational guidance, making consistent use difficult), (4) training and readiness (e.g. technologies were sometimes deployed before training was available,), (5) usability and accessibility (e.g, systems were described as unintuitive, requiring excessive steps to complete simple tasks) and (6) communication and technical dependency (e.g. communication occasionally broke down when digital tools failed). A summary of these issues by respondents' country is presented in Figure 2.

Digital literacy and technical skills emerged as significant challenges in the USA (40.23%, *n* = 140) followed by Australia (21.82%, *n* = 12), Switzerland and the United Kingdom (6.82%, *n* = 6). No issues in this category were reported in Finland. Technical barriers and connectivity issues were notable in Australia (20%, *n* = 11), the United Kingdom (18.18%, *n* = 16) and Switzerland (10.71%, *n* = 3), while Finland reported none. Despite reporting a wide range of challenges, respondents made minimal reference to issues concerning data protection, privacy or legal and ethical responsibilities when using digital technologies. Such concerns appeared only sporadically in the free‐text responses and did not form a separate analytical category, indicating that information governance considerations were not commonly raised by participants across countries.

Adoption and organizational challenges were reported in all six countries, with Finland (76.92%, *n* = 30) and the United Kingdom (45.45%, *n* = 40) reporting the most issues, while the USA had fewer (10.34%, *n* = 36). Switzerland's training and readiness issues were unique (28.57%, *n* = 8) as these were not reported by other countries. Usability and accessibility challenges were reported primarily by the United Kingdom (27.27%, *n* = 24) and Australia (16.36%, *n* = 9), with no issues in this domain from the USA, Finland, New Zealand or Switzerland. Communication and technical dependency were most problematic in New Zealand, accounting for 57.58% (*n* = 19) of all reported issues.

## 4 Discussion

### 4.1 Summary of Key Findings

This study combines results from six countries across three continents to provide an international comparison of nurses’ experiences of the adoption and use of technologies during the COVID‐19 pandemic, aiming to identify lessons learned that may inform future technology adoption in public healthcare crises. With a total of 12 categories, the most reported technologies were related to *care and patient information* and *monitoring*. The usability of all described technologies was rated at the benchmark for average usability, with Switzerland and New Zealand reporting the highest overall scores. Finally, the most common challenges related to technology adoption cross‐nationally included *Digital Literacy and Skills* and *Communication and Technical Dependency*, with country‐specific variations in both the results and their emphases.

### 4.2 Relation of the Study Findings to the Existing Body of Knowledge

The countries participating in this study have attained at least a moderate degree of digital maturity, with most currently situated at more advanced development phases (World Health Organization 2025). Despite these advancements, our findings reveal notable cross‐national variation in the usability of different technology categories. Given that many of the reported technologies (such as electronic health record systems, telehealth and monitoring tools) are broadly similar across settings, these differences suggest that some countries have been more successful in optimizing system integration, workflow alignment and user experience. Drawing on the NASSS framework (Greenhalgh et al. 2017), cross‐national comparison is essential not only for identifying context‐specific complexities, but also for pinpointing shared factors that can inform improvements in system design, infrastructure and overall usability.

The differences observed in usability and adoption challenges indicate that countries with more established and better‐integrated digital infrastructures were better positioned to deploy usable technologies rapidly during the pandemic. This underlines the importance of strengthening baseline digital infrastructure, interoperability and organizational readiness as part of preparedness planning. Investing in these foundations enables health systems to respond more effectively in future crises, ensuring that technologies can be implemented quickly, safely and with consistent usability across settings. Moreover, comparing countries where particular systems performed well or poorly allows the identification of best practices and transferable lessons to support more effective and user‐centered digital health adoption globally.

The WHO operational framework for disaster resilience illustrates how healthcare systems respond to major disruptions. This framework highlights the relationship between a system's context, its stresses (such as the COVID‐19 pandemic) and its capacity to manage, respond to and ultimately recover from these disturbances (World Health Organization 2015). Our findings highlight that nurses experienced variability in the categories of technologies introduced and how usable or useful those systems were in supporting care. For example, specific categories of technologies, such as digital care pathways and patient portals (74.56) and monitoring devices (72.23), showed consistently higher usability across countries. Digital care pathways and patient portals received high scores, especially in the United Kingdom, suggesting that users generally well received these technologies (Carini et al. 2021). Conversely, technologies with unclear purposes had the lowest usability scores (56.17), followed by data management infrastructure (61.38), highlighting potential usability issues such as poor user interface design, inadequate support, or insufficient integration with existing workflows. This aligns with the prior literature on nursing leadership that reports issues with information management systems across settings (Peltonen et al. 2018, Saranto et al. 2023). Moreover, the high usability scores in specific categories indicate that understanding best practices from countries like Switzerland and New Zealand could provide valuable insights into implementing systems and improving their usability across countries. This is supported by research showing that pioneering work in contact tracing (Amann et al. 2021) and the digitalization of information systems led to a better ability to provide daily updates on the number of confirmed Covid‐19 cases, deaths and tests as well as more detailed data such as the impact of co‐morbidities on case severity in Switzerland (Desson et al. 2020).

### 4.3 Implications for Practice and Policy

These cross‐national similarities and differences provide transferable insights that can guide policy‐level decision‐making to strengthen digital preparedness in future public health crises. The participants in this study reported a variety of challenges related to technology adoption, including digital literacy and skills, training and readiness, adoption and organizational challenges, and technical barriers. It is important to understand the impact of these contextual factors on usability in order to prepare for future public health crises.

Understanding contextual differences is crucial for successful adoption and user satisfaction (Tang et al. 2018), and we need insight into whether this has been successful or not when services must be flexible and adapt quickly to maintain care. Prior research has shown that end users are not sufficiently involved in the development of implemented technologies (Silva et al. 2024, Martikainen et al. 2020). Failing to involve them throughout the technology development and implementation process significantly impacts how systems respond to different users’ needs in clinical practice and how they are received (Keizer et al. 2020). A one‐size‐fits‐all approach may not function and tailored solutions addressing the specific needs of users in different settings are needed. Addressing these issues may potentially improve the overall effectiveness of systems and service delivery. Identifying the exact reasons for low scores in some countries could help improve the usability and effectiveness of these technologies overall and support resilience building for future public health crises, as differences in usability scores may be influenced by issues such as training provided (Rossetto et al. 2023), system design (Carayon and Hoonakker 2019), technology maturity and how technology can be integrated into existing workflows (Herranz et al. 2023). Continuous development is a key strategy in responding to the needs of a rapidly evolving health setting (World Health Organization 2015). One added value of this study lies in its potential to inform the future preparedness of the nursing workforce for managing similar public health crises where rapid responses are needed.

In this study, challenges related to digital literacy and skills were prevalent across almost all countries, significantly affecting participants in the USA and Australia. Despite the growing integration of digital technologies in modern healthcare, informatics and digital health competencies essential for safe nursing practice remain suboptimal (Dowding et al. 2023, Livesay et al. 2023). This study argues for the need to make high‐quality informatics education and training mandatory at the organizational level to ensure the workforce is prepared for future crises and can adapt quickly when required. This is particularly important in the absence of mandatory informatics and digital technology training in pre‐registration nursing programs.

The United Nations advises countries to strengthen their response to disease and prepare for future public health crises and other global threats (United Nations 2023). Based on our findings, we can make some initial suggestions of areas where nurse leaders should focus to ensure their health system and the nursing workforce are prepared for future public health crises, including potential pandemics. First, the adoption and organizational challenges and technical barriers described by the participants imply that healthcare organizations need to identify and invest in appropriate infrastructure to support digital technology implementation. Second, the usability and accessibility challenges expressed by the participants, combined with the average SUS score suggesting usability below the acceptable level, highlight the need to include end users in the technology development. Nurses need to be included in procuring and implementing future digital technologies to prepare for enhanced uptake and widespread adoption. Finally, challenges related to digital literacy and skills underline the importance of proper investment in informatics education and training for the nursing workforce for successful digital transformation. The findings also highlight the importance of identifying global ‘best practices’ to inform rapid technology implementation in healthcare to better prepare for future global public health threats.

### 4.4 Strengths and Limitations

Study limitations include self‐reported data, a small sample size for specific regions, the limitation to data collection in high‐income countries, possible misclassification of technologies and variability in response scales. Further, the disparity in participant numbers across countries reflecting both the reach of individual professional networks as well as the overall size of the nursing workforce within each participating country increases the risk of sampling bias in this study. Despite normalization, the variability in the initial response scales may have introduced inconsistencies or imprecision in the SUS scores, potentially affecting the accuracy of cross‐country comparisons.

### 4.5 Future Research

Although respondents described a wide range of adoption challenges, including digital literacy, organizational readiness, usability issues and technical barriers, there was minimal reference to data protection, privacy or legal‐ethical responsibilities in the free‐text responses. This relative absence is striking given the centrality of information governance in digital health. Future studies should therefore examine nurses’ current understanding of legal, ethical and social implications of digital technologies, and explore whether gaps in awareness, training or perceived relevance may explain why these concerns were not raised in the present dataset. Hence, future work could explore nurses’ current understanding of social, legal and ethical perspectives of applying technologies to nursing and care.

## 5 Conclusion

It is vital to learn from nurses’ experiences of the rapid adoption and implementation of digital technologies during the COVID‐19 pandemic, to inform preparedness for future public health crises. Our study found that nurses were requested to use technologies that had system usability issues, which varied significantly between technology categories and countries. Also, adoption challenges were seen across settings regardless of differences in the societies’ digital maturity. This underscores the importance of cross‐country learning to enhance system design, infrastructure and overall usability. The analysis of system usability highlights strengths and needs for improvement in the usability of technologies implemented in nursing. Healthcare providers and technology developers can better support the effective use of technologies by focusing on user‐centered design, localized tailoring and targeted developments in specific categories and regions.

While adopting digital technologies during the COVID‐19 pandemic has brought numerous benefits, it is not without challenges. Some key considerations include ensuring equitable access to digital technologies and promoting health literacy among patients and healthcare providers. Disparities in access to internet connectivity, devices and digital literacy can hinder the effective implementation of these technologies, particularly among underserved populations. Further, the increased reliance on digital technologies necessitates robust privacy and security measures to protect patient information. While challenges still occur, such as technological infrastructure and competency concerns, the pandemic did boost an opportunity for more long‐term integration of technologies into practice. The results of this study may be used for international comparisons and the development of education and practice to better support nurses in future technology adoption.

## Funding

The Burdett Trust for Nursing funded the UK study and the Finnish Work Environment Fund for the Finnish study.

## Ethics Statement

Each country sought ethical approval for local data collection in accordance with its specific regulatory requirements.

## Conflicts of Interest

The authors declare no conflicts of interest.

## Data Availability

The authors have nothing to report.

## References

[phn70073-bib-0001] Abell, B. , S. Naicker , D. Rodwell , et al. 2023. “Identifying Barriers and Facilitators to Successful Implementation of Computerized Clinical Decision Support Systems in Hospitals: A NASSS Framework‐Informed Scoping Review.” Implementation Science 18, no. 1: 32. 10.1186/s13012-023-01287-y. 37495997 PMC10373265

[phn70073-bib-0002] Amann, J. , J. Sleigh , and E. Vayena . 2021. “Digital Contact‐tracing During the Covid‐19 Pandemic: An Analysis of Newspaper Coverage in Germany, Austria, and Switzerland.” PLOS ONE 16, no. 2: e0246524. 10.1371/journal.pone.0246524. 33534839 PMC7857553

[phn70073-bib-0003] Anderson, H. , A. Scantlebury , P. Galdas , and J. Adamson . 2024. “Remote and Technology‐Mediated Working During the COVID‐19 Pandemic: A Qualitative Exploration of the Experiences of Nurses Working in General Practice (the GenCo Study).” Journal of Advanced Nursing 80, no. 4: 1592–1606. 10.1111/jan.15921. 37909600

[phn70073-bib-0004] Atique, S. , J. R Bautista , L. J Block , et al. 2020. “A Nursing Informatics Response to COVID‐19: Perspectives From Five Regions of the World.” Journal of Advanced Nursing 76, no. 10: 2462–2468. 10.1111/jan.14417. 32420652 PMC7276900

[phn70073-bib-0005] Blöndal, K. , S. H Sverrisdóttir , A. Hafberg , et al. 2022. “Confronting the Unknown‐Nursing Surveillance of COVID‐19‐Infected Patients Through Remote Telephone Calls and in an On‐Site Urgent Clinic.” Journal of Advanced Nursing 78, no. 11: 3782–3794. 10.1111/jan.15355. 35975315 PMC9538875

[phn70073-bib-0006] Bokolo, A. J. 2021. “Application of Telemedicine and eHealth Technology for Clinical Services in Response to COVID‑19 Pandemic.” Health and Technology 11, no. 2: 359–366. 10.1007/s12553-020-00516-4. 33469474 PMC7808733

[phn70073-bib-0007] Brooke, J. 1996. “SUS: A ‘Quick and Dirty’ Usability Scale.” In Usability Evaluation in Industry, edited by P. W. Jordan , B. Thomas , B. A. Weerdmeester , and I. L. McClelland . CRC Press, pp. 189–194.

[phn70073-bib-0008] Cadel, L. , M. Marcinow , J. Sandercock , et al. 2021. “A Scoping Review of Patient Engagement Activities During COVID‐19: More Consultation, Less Partnership.” PLOS ONE 16, no. 9: e0257880. 10.1371/journal.pone.0257880. 34587175 PMC8480845

[phn70073-bib-0009] Carayon, P. , and P. Hoonakker . 2019. “Human Factors and Usability for Health Information Technology: Old and New Challenges.” Yearbook of Medical Informatics 28, no. 1: 71–77. 10.1055/s-0039-1677907. 31419818 PMC6697515

[phn70073-bib-0010] Carini, E. , L. Villani , A. M Pezzullo , et al. 2021. “The Impact of Digital Patient Portals on Health Outcomes, System Efficiency, and Patient Attitudes: Updated Systematic Literature Review.” Journal of Medical Internet Research 23, no. 9: e26189. 10.2196/26189. 34494966 PMC8459217

[phn70073-bib-0011] Collins, E. , and M. Honey . 2021. “Access as an Enabler and an Obstacle to Nurses' use of ICT During the COVID‐19 Pandemic: Results of a National Survey.” Nursing Praxis in Aotearoa New Zealand 37, no. 3: 62–70. 10.36951/27034542.2021.036.

[phn70073-bib-0012] Desson, Z. , L. Lambertz , J. W Peters , M. Falkenbach , and L. Kauer . 2020. “Europe's Covid‐19 Outliers: German, Austrian and Swiss Policy Responses During the Early Stages of the 2020 Pandemic.” Health Policy and Technology 9, no. 4: 405–418. 10.1016/j.hlpt.2020.09.003. 33520639 PMC7834269

[phn70073-bib-0013] Dowding, D. , S. Skyrme , R. Randell , L. Newbould , M. Faisal , and N. Hardiker . 2023. “Researching Nurses' use of Digital Technology During the COVID‐19 Pandemic.” Nursing Standard 38, no. 7: 63–68. 10.7748/ns.2023.e12013. 37157913

[phn70073-bib-0014] Dykes, S. , and C. H Chu . 2021. “Now More Than Ever, Nurses Need to be Involved in Technology Design: Lessons From the COVID‐19 Pandemic.” Journal of Clinical Nursing 30, no. 7‐8: e25–e28. 10.1111/jocn.15581. 33289230 PMC7753642

[phn70073-bib-0015] Elo, S. , and H. Kyngäs . 2008. “The Qualitative Content Analysis Process.” Journal of Advanced Nursing 62, no. 1: 107–115. 10.1111/j.1365-2648.2007.04569.x. 18352969

[phn70073-bib-0016] Greenhalgh, T , and S. Abimbola . 2019. “The NASSS Framework—A Synthesis of Multiple Theories of Technology Implementation.” Studies in Health Technology and Informatics 263: 193–204. 10.3233/SHTI190123. 31411163

[phn70073-bib-0017] Greenhalgh, T. , J. Wherton , C. Papoutsi , et al. 2017. “Beyond Adoption: A New Framework for Theorizing and Evaluating Nonadoption, Abandonment, and Challenges to the Scale‐Up, Spread, and Sustainability of Health and Care Technologies.” Journal of Medical Internet Research 19, no. 11: e367. 10.2196/jmir.8775. 29092808 PMC5688245

[phn70073-bib-0018] Grindle, K. R. 2021. “Impact of Technology on Community Nursing During the Pandemic.” British Journal of Community Nursing 26, no. 3: 110–115. doi: 10.12968/bjcn.2021.26.3.110. 33719559

[phn70073-bib-0019] Hargreaves, L. , P. Zickgraf , N. Paniagua , T. L Evans , and L. Radesi . 2021. “COVID‐19 Pandemic Impact on Nursing Student Education: Telenursing With Virtual Clinical Experiences.” Sage Open Nursing 7: 23779608211044618. 10.1177/23779608211044618. 34692998 PMC8529906

[phn70073-bib-0020] Heath, J. , M. Moran , and A. Dowrick . 2024. “Examining Qualitative Cross‐Country Comparative Analysis in Health: Reflective Insights and Methodological Considerations.” SSM ‐ Qualitative Research in Health 5: 100416. 10.1016/j.ssmqr.2024.100416.

[phn70073-bib-0021] Herranz, C. , L. Martín‐Moreno Banegas , F. Dana Muzzio , A. Siso‐Almirall , J. Roca , and I. Cano . 2023. “A Practice‐Proven Adaptive Case Management Approach for Innovative Health Care Services (Health Circuit): Cluster Randomized Clinical Pilot and Descriptive Observational Study.” Journal of Medical Internet Research 25: e47672. 10.2196/47672. 37314850 PMC10337458

[phn70073-bib-0022] James, H. M. , C. Papoutsi , J. Wherton , T. Greenhalgh , and S. E Shaw . 2021. “Spread, Scale‐Up, and Sustainability of Video Consulting in Health Care: Systematic Review and Synthesis Guided by the NASSS Framework.” Journal of Medical Internet Research 23, no. 1: e23775. 10.2196/23775. 33434141 PMC7837451

[phn70073-bib-0023] Jedwab, R. M. , E. Manias , B. Redley , N. Dobroff , and A. M Hutchinson . 2023. “Impacts of Technology Implementation on Nurses' work Motivation, Engagement, Satisfaction and Well‐Being: a Realist Review.” Journal of Clinical Nursing 32, no. 17‐18: 6037–6060. 10.1111/jocn.16730. 37082879

[phn70073-bib-0024] Jeon, E. , L. M Peltonen , L. J Block , et al. 2024. “Technological Challenges and Solutions in Emergency Remote Teaching for Nursing: an International Cross‐Sectional Survey.” Healthcare Informatics Research 30, no. 1: 49–59. 10.4258/hir.2024.30.1.49. 38359849 PMC10879829

[phn70073-bib-0025] Keizer, J. , N. B Jong , N. A Naiemi , and J. E van Gemert‐Pijnen . 2020. “Persuading From the Start: Participatory Development of Sustainable Persuasive Data‐Driven Technologies in Healthcare.” In Persuasive Technology. Designing for Future Change: 15th International Conference on Persuasive Technology, PERSUASIVE 2020, Aalborg, Denmark, April 20–23, 2020, Proceedings (pp. 113–125). Springer International Publishing.

[phn70073-bib-0026] Lewis, J. R. , and J. Sauro . 2018. “Item Benchmarks for the System Usability Scale.” Journal of Usability Studies 13, no. 3.

[phn70073-bib-0027] Livesay, K. , S. Petersen , R. Walter , L. Zhao , K. Butler‐Henderson , and R. Abdolkhani . 2023. “Sociotechnical Challenges of Digital Health in Nursing Practice during the COVID‐19 Pandemic: National Study.” JMIR Nursing 6: e46819. 10.2196/46819. 37585256 PMC10468699

[phn70073-bib-0028] Logsdon, M. C. 2022. “Technology Use During COVID‐19 Pandemic: Future Implications for Nursing and Health Care.” Computers, Informatics, Nursing 40, no. 5: 291–292. 10.1097/CIN.0000000000000906. PMC9093227 35523225

[phn70073-bib-0029] Martikainen, S. , J. Kaipio , and T. Lääveri . 2020. “End‐User Participation in Health Information Systems (HIS) Development: Physicians' and Nurses' Experiences.” International Journal of Medical Informatics 137: 104117. 10.1016/j.ijmedinf.2020.104117. 32179254

[phn70073-bib-0030] Meehan, B. , and M. Honey . 2020. “Hot Tips to Assist a Virtual Patient Assessment in Uncertain Times.” Kai Tiaki Nursing New Zealand 26, no. 3: 18–19.

[phn70073-bib-0031] Meinert, E. , A. Alturkistani , D. Brindley , P. Knight , G. Wells , and N. De Pennington . 2018. “Weighing Benefits and Risks in Aspects of Security, Privacy and Adoption of Technology in a Value‐Based Healthcare System.” BMC Medical Informatics and Decision Making 18: 100. 10.1186/s12911-018-0700-0. 30424753 PMC6234649

[phn70073-bib-0032] O'Leary, L. , S. Erikainen , L. M Peltonen , W. Ahmed , M. Thelwall , and S. O'Connor . 2022. “Exploring Nurses' Online Perspectives and Social Networks During a Global Pandemic COVID‐19.” Public Health Nursing 39, no. 3: 586–600. 10.1111/phn.12994. 34687078 PMC8661865

[phn70073-bib-0033] Pang, T. Y. , T. K Lee , and M. Murshed . 2023. “Towards a New Paradigm for Digital Health Training and Education in Australia: Exploring the Implication of the Fifth Industrial Revolution.” Applied Sciences 13, no. 11: 6854.

[phn70073-bib-0034] Patel, H. , A. Hassell , B. Cyriacks , B. Fisher , W. Tonelli , and C. Davis . 2022. “Building a Real‐Time Remote Patient Monitoring Patient Safety Program for COVID‐19 Patients.” American Journal of Medical Quality 37, no. 4: 342–347. 10.1097/JMQ.0000000000000046. 35213860 PMC9241558

[phn70073-bib-0035] Peltonen, L. M. , K. Junttila , and S. Salanterä . 2018. “Nursing Leaders' Satisfaction With Information Systems in the Day‐to‐Day Operations Management in Hospital Units.” Studies in Health Technology and Informatics 250: 203–207. 29857436

[phn70073-bib-0036] Pozzi, F. , F. Manganello , M. Passarelli , D. Persico , and M. Romagnoli . 2023. “Collaborative Approaches in Online Nurse Education: A Systematic Literature Review.” Electronic Journal of e‐Learning 21, no. 3: 121–140.

[phn70073-bib-0037] Rossetto, F. , F. Borgnis , S. Isernia , et al. 2023. “System Integrated Digital Empowering and TeleRehabilitation to Promote Patient Activation and Well‐Being in Chronic Disabilities: A Usability and Acceptability Study.” Frontiers in Public Health 11: 1154481. 10.3389/fpubh.2023.1154481. 37250091 PMC10214955

[phn70073-bib-0038] Santos, M. D. , C. Roman , M. A F. Pimentel , et al. 2021. “A Real‐Time Wearable System for Monitoring Vital Signs of COVID‐19 Patients in a Hospital Setting.” Frontiers in Digital Health 3: 630273. 10.3389/fdgth.2021.630273. 34713102 PMC8521865

[phn70073-bib-0039] Saranto, K. , S. Koponen , T. Vehko , and E. Kivekäs . 2023. “Nurse Managers' Opinions of Information System Support for Performance Management: A Correlational Study.” Methods of Information in Medicine 62, no. S 01: e63–e72. 10.1055/a-1978-9727. 36379471 PMC10306445

[phn70073-bib-0040] Sauro, J. , and J. Lewis . 2016. Quantifying the User Experience: Practical Statistics for User Research. 2nd ed. Elsevier/Morgan Kaufmann.

[phn70073-bib-0041] Sheikh, A. , M. Anderson , S. Albala , et al. 2021. “Health Information Technology and Digital Innovation for National Learning Health and Care Systems.” Lancet Digital Health 3, no. 6: e383–e396. 10.1016/S2589-7500(21)00005-4. 33967002

[phn70073-bib-0042] Shin, H. D. , E. Hamovitch , E. Gatov , et al. 2025. “The NASSS (Non‐Adoption, Abandonment, Scale‐Up, Spread and Sustainability) Framework use Over Time: A Scoping Review.” PLOS Digit Health 4, no. 3: e0000418. 10.1371/journal.pdig.0000418. 40096260 PMC11913280

[phn70073-bib-0043] Silva, J. , A. Araújo , F. Coutinho , and A. Silva . 2024. “Are End‐Users Participating in the Life Cycle of Healthcare Application Development? An Analysis of the Opportunities and Challenges of the use of HCI Techniques in the Healthcare Sector.” BIOSTEC 2: 789–796.

[phn70073-bib-0044] Tang, T. , M. E Lim , E. Mansfield , A. McLachlan , and S. D Quan . 2018. “Clinician User Involvement in the Real World: Designing an Electronic Tool to Improve Interprofessional Communication and Collaboration in a Hospital Setting.” International Journal of Medical Informatics 110: 90–97. 10.1016/j.ijmedinf.2017.11.011. 29331258

[phn70073-bib-0045] United Nations . The World Must be Ready to Respond to the Next Pandemic [Internet]. 2023. [cited 2024 Nov 1]. https://news.un.org/en/story/2023/05/1136912#:~:text=Although%20COVID%2D19%20may%20no,said%20on%20Monday%20in%20Geneva.

[phn70073-bib-0046] van Calis, J. F E. , K. E Bevelander , A. W C. van der Cruijsen , G. L Leusink , and J. Naaldenberg . 2023. “Toward Inclusive Approaches in the Design, Development, and Implementation of eHealth in the Intellectual Disability Sector: Scoping Review.” Journal of Medical Internet Research 25: e45819. 10.2196/45819. 37252756 PMC10265410

[phn70073-bib-0047] Vasilica, C. , M. Wynn , D. Davis , K. Charnley , and L. Garwood‐Cross . 2023. “The Digital Future of Nursing: Making Sense of Taxonomies and Key Concepts.” British Journal of Nursing 32, no. 9: 442–446. doi: 10.12968/bjon.2023.32.9.442. 37173087

[phn70073-bib-0048] Wieben, A. M. , R. L Walden , B. G Alreshidi , et al. 2023. “Data Science Implementation Trends in Nursing Practice: A Review of the 2021 Literature.” Applied Clinical Informatics 14, no. 3: 585–593. 10.1055/a-2088-2893. 37150179 PMC10411069

[phn70073-bib-0049] World Health Organization . Operational Framework for Building Climate Resilient Health Systems [Internet]. 2015. [cited 2025 Mar 5]: https://www.who.int/publications/i/item/operational‐framework‐for‐building‐climate‐resilient‐health‐systems.

[phn70073-bib-0050] World Health Organization . Global Digital Health Monitor [Internet]. 2025. [cited 2025 Aug 26]: https://data.who.int/dashboards/gdhm/overview.

